# To sling or not to sling? Impact of intraoperative sling procedures during radical prostatectomy on postoperative continence outcomes: A systematic review and meta‐analysis

**DOI:** 10.1002/bco2.67

**Published:** 2021-01-17

**Authors:** Eunice Lim, Scott Leslie, Ruban Thanigasalam, Daniel Steffens

**Affiliations:** ^1^ RPA Institute of Academic Surgery (IAS) Royal Prince Alfred Hospital University of Sydney Sydney NSW Australia; ^2^ Concord Institute of Academic Surgery Concord Repatriation General Hospital Sydney NSW Australia; ^3^ Faculty of Medicine and Health University of Sydney Sydney NSW Australia; ^4^ Surgical Outcomes Research Centre (SOuRCe) Sydney NSW Australia

**Keywords:** incontinence, intraoperative slings, prostatectomy

## Abstract

**Purpose:**

This systematic review and meta‐analysis investigates the efficacy of intraoperative sling procedures in reducing postprostatectomy urinary incontinence compared to having no slings.

**Methods:**

A comprehensive search of PubMed, Medline, Embase, and the Cochrane library from inception to November 2020 was performed. Risk of bias was assessed using the Cochrane Risk of Bias tool for randomized studies and Newcastle‐Ottawa Scale for nonrandomized studies. The GRADE approach was used for critical appraisal of evidences and meta‐analyses were conducted using random‐effects models.

**Results:**

Ten studies were included (n = 1,447). Quality of evidence ranged from moderate to very low. Sling procedure was generally favorable for short‐term continence outcomes, although discrepancies exist due to variability in continence definition. Sling procedure resulted in reduced urinary pad weight at 1 month postoperatively (MD: 21.55; 95%CI: 12.58 to 30.52). Patient‐reported questionnaires were also favorable for the sling group for up to 3 months (IPSS; (MD: 1.44; 95%CI: 0.14 to 2.74), ICIQ‐SF; (MD: 2.25; 95%CI: 1.26 to 3.24), EPIC‐U; (MD: 5.30; 95%CI: 1.12 to 9.39)) postoperatively. Sling procedure improved the number of continent patients at 1 month with continence definition of zero pad use/day (RR:1.41; 95%CI: 1.10 to 1.83) but not with the definition of ≤ 1pad/day. Similarly, it reduced the time to continence with the ≤ 1 pad/day definition (MD: 0.5; 95%CI: 0.1 to 0.9) but not with the zero pad/day definition.

**Conclusion:**

The current literature suggests that intraoperative sling procedures during radical prostatectomy may promote early return of continence compared to having no sling, however, there are no long‐term differences.

## 1 INTRODUCTION

Prostate cancer is the second most common malignancy in men worldwide, with 1.3 million new cases in 2018.^1^ Radical prostatectomy is an effective and commonly performed treatment option for localized prostate cancer.^2^ However, urinary incontinence remains a major adverse event following radical prostatectomy, with 12 months postoperative urinary incontinence rate ranging from 4% to 31%.^3^ Urinary incontinence can have a severe impact on the patient's quality of life, and thus, remains a deterrent for many patients when deciding treatment for their prostate cancer.^3^

Multiple etiologies have been proposed for the development of postoperative incontinence with the major factor attributed to intraoperative damage of the urethral sphincter with accompanying intrinsic sphincter deficiency.^4^ Other possible causes include detrusor over and under‐activity, bladder outlet obstruction due to anastomotic strictures and damage to pelvic nerves that potentially supply the sphincter mechanism. As such, several intraoperative techniques aiming to reduce postoperative urinary incontinence have been described, including surgical techniques to preserve anatomical structures (ie, bladder neck, neurovascular bundle, or puboprostatic ligaments) and different surgical approaches including anterior and posterior reconstruction and the Retzius‐sparing technique.^5^, ^6^ However, the role of these intraoperative techniques in reducing incontinence remains unclear; with many of these techniques not externally validated in a multicenter setting.^7^, ^8^, ^9^

Recent studies suggest that the use of intraoperative suburethral sling techniques during prostatectomy may reduce the incidence of postoperative urinary incontinence.^10^ Sling suspension technique involves the placement of a sling to support the proximal urethra and bladder neck to provide compressive force on the urethra, increase the functional length of the urethra and maintain adequate urethral closure.^5^ Sling techniques are commonly used in the setting of *postoperative* urinary incontinence when noninvasive therapies such as pelvic floor muscle training and pharmacologic treatments fail.^11^ Performing a sling procedure *intra‐operatively* at the time of prostatectomy may prevent the development of urinary complications, improving postoperative quality of life and reducing overall cost by minimizing the likelihood of patients having to undergo secondary surgery for incontinence following radical prostatectomy.

Different surgical techniques and sling materials have been explored with varying results. Therefore, the purpose of this study is to conduct a systematic review and meta‐analysis to evaluate the effectiveness of intraoperative slings compared with no sling procedure in reducing postoperative urinary incontinence and complication rates in patients undergoing radical prostatectomy.

## 2 MATERIALS AND METHODS

This systematic review and meta‐analysis was registered with PROSPERO (https://www.crd.york.ac.uk/PROSPERO/, CRD42017078878) prior to commencement of the review. The protocol followed the methods recommended by the Cochrane Handbook for Systematic Reviews of Interventions^12^ and was written in accordance with PRISMA‐P (Preferred Reporting Items for Systematic Reviews and Meta‐Analysis Protocols) statement.^13^

### 2.1 Search strategy

A sensitive search was performed from inception to November 2020 in MEDLINE via OVID, EMBASE via OVID, PubMed (www.ncbi.nlm.nih.gov/pubmed) and Cochrane Central Register Clinical Trials (CENTRAL) via the Cochrane Library, using key words related to “prostate cancer,” “prostatectomy,” “sling,” and “urinary incontinence” (Table S1). No language restrictions were applied. In addition, reference lists of all included studies were screened.

One reviewer inspected the titles and abstracts of all identified studies to generate a list of potentially eligible studies (EL). Full‐text articles were reviewed by two review authors for eligibility (EL and DS). Consensus between the two reviewers was used to resolve any disagreement.

### 2.2 Inclusion and exclusion criteria

The studies included in this review reported data on localized prostate cancer patients who underwent radical prostatectomy at any age. Study types included randomized controlled trials and nonrandomized comparative studies.

Exclusion criteria includes animal studies, conference abstractions or poster publications, and descriptive commentaries.

### 2.3 Outcome measures

The main outcomes of interest were urinary incontinence and other adverse events or complications. Length of hospital stay, length of operation, quality of life, and cost were also investigated. Postoperative outcome data were extracted at 4 time points: 1, 3, 6, and 12 months.

### 2.4 Data extraction

Two review authors used a pre‐piloted data extraction sheet to extract data from the included studies (EL and DS). Consensus between the two reviewers were used to resolve any disagreement. An attempt was made to contact authors from studies where data were unclear or not available in the published manuscript.

For each included study the following data were extracted: sample characteristics (country, study design, surgical procedure, sling position, sling material, age, body mass index, prostate specific antigen (PSA)), name of sling procedure used, and all outcome measures from all time points.

### 2.5 Risk of bias assessment and strength of the evidence

Cochrane Collaboration's tool was used to assess the methodological quality of randomized controlled trials.^12^ Each study was assessed as having high, low, or unclear risk of bias by two reviewers (EL and DS). Methodological quality of comparative studies was assessed via the Newcastle‐Ottawa Scale (NOS), which was endorsed by the Cochrane handbook for quality appraisal of observational studies.^14^, ^15^ Each study was given a score between zero and nine, by considering three factors that consist of nine items in total: (1) Selection of study groups, (2) Comparability and (3) Outcome of interest.^14^ Our review considered a study with a score of ≥ 7 as having high quality and low risk of bias, as there are no established standardized criteria for the interpretation of the NOS scores currently. Grading of Recommendations Assessment, Development and Evaluation (GRADE) approach was used to assess strength of evidence from high to very‐low‐quality for each outcomes.^16^

### 2.6 Statistical analysis

For dichotomous outcomes, we extracted the number of patients in each group who experienced the outcome of interest and the number of patients assessed at endpoint in each treatment arm at the end of the prespecified follow‐up, in order to estimate a relative risk and its 95% confidence interval. For continuous outcomes, we extracted the final value and standard deviation of the outcome of interest and the number of patients assessed at the endpoint in each treatment arm at the end of follow‐up. Where appropriate, we calculated the mean difference and 95% confidence interval.

Outcome measures from individual trials were combined through a meta‐analysis where possible using a random‐effects model, via the Comprehensive Meta‐Analysis software.^17^ When a meta‐analysis was not possible, results were described qualitatively.

Included studies were grouped by outcome measures (eg, continence defined as zero pad/day), followed by time frame (1, 3, 6, and 12 months) to provide a homogenous subset for meta‐analysis. Time points (1, 3, 6, and 12 months) were selected based on the available results. Studies included in the meta‐analysis were ordered chronologically.

## 3 RESULTS

### 3.1 Literature search

**FIGURE 1:**
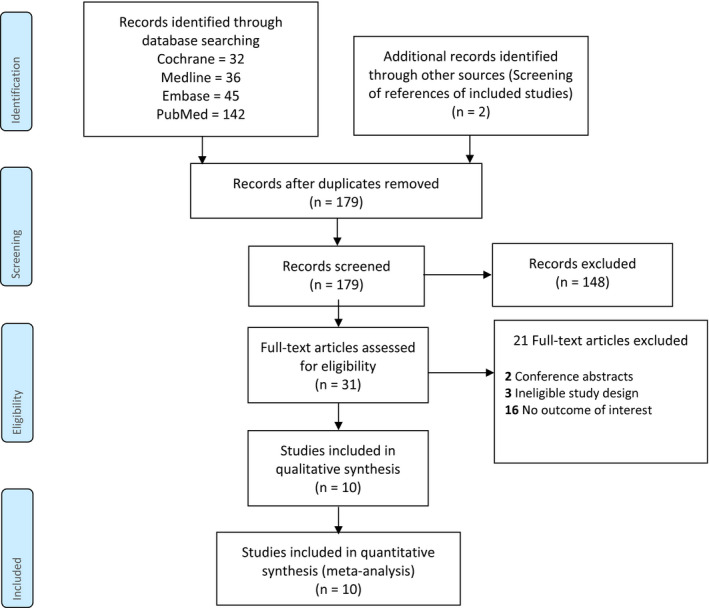
PRISMA Flow diagram of review process

The search identified 179 citations after the removal of duplicates. Following the elimination of irrelevant references, 31 full‐text articles were screened for eligibility. About 21 articles were excluded due to the following reasons: conference abstracts (n = 2); ineligible study design (n = 3); and no outcome of interest (n = 16). Therefore, 10 studies ^10^, ^26^ were included in this systematic review (n = 1,447) (Figure 1).

### 3.2 Study characteristics

Included trials consisted of five RCTs^18^, ^19^, ^20^, ^21^, ^22^ and five retrospective cohort studies.^10^, ^23^, ^24^, ^25^, ^26^ All slings were positioned on the bladder neck, and sling material used included rectus fascia (n = 3), small intestinal submucosa (n = 2), vas deferens (n = 3), Denonvilliers’ fascia (n = 1), median umbilical ligament (n = 1), and retrotrigonal muscular layer (n = 1). All slings identified and included in the study were nonsynthetic slings (Table 1).

**TABLE 1:** Characteristics of the included studies

Author, year	Country where study was performed	Study design	Procedure	Sling position	Sling material	PSA (SD)	Age, years (SD)	BMI (SD)	Intervention (Baseline N/Follow‐up N)	Control (Baseline N/Follow‐up N)
Altinova, 2009	Turkey	RCT	RRP	Bladder neck	Rectus fascia/1‐prolene	12.96 (10.39)	63.47 (7.23)	NR	Anterior Rectus Fascial Sling (40/40)	No sling (46/46)
Bahler, 2016	US	RCT	RARP	Bladder neck	Small intestinal submucosa	6.21 (3.47)	60.7 (6.6)	29.7 (5.16)	Cook Biodesign Surgisis urethral sling (74/60)	No Sling (73/55)
Westney, 2006	US	Retrospective Cohort Study	RRP	Bladder neck	Rectus fascia/0‐prolene	NR	NR	NR	Suburethral sling (49/46)	No sling (122/89)
Nguyen, 2017	US	RCT	RARP	Bladder neck	Vas deferens/vicryl	6.75 (1.44)	63.35 (6.53)	NR	Retropubic urethral sling (93/82)	No sling (99/86)
Cestari, 2015	Italy	RCT	RARP	Bladder neck	Denonvilliers’ fascia/2‐0 vicryl	8.63 (3.83)	63 (9.0)	25.9	Retropubic suburethral autologous sling (30/30)	No sling (30/30)
Kojima, 2014	Japan	RCT	RARP	Bladder neck	Vas deferens/3‐0 v‐loc	7.35 (1.09)	65.7 (2.27)	23.4	Bladder neck sling (27/27)	No sling (30/30)
Jorion, 1997	Belgium	Retrospective Cohort Study	RRP	Bladder neck	Rectus fascia	NR	64 (5.92)	NR	Rectus Fascial Sling (30/30)	No sling (30/30)
Punnen, 2014	US	Retrospective Cohort Study	RARP	Bladder neck	Median umbilical ligament or vas deferens/ 3‐0 v‐loc	5.93 (6.43)	NR	NR	Retropubic urethral sling (156/153)	No sling (81/78)
Jones, 2005	US	Retrospective Cohort study	RRP	Bladder neck	Porcine small intestine submucosa/polyglactin mesh	NR	60.15 (7.28)	NR	Stratisis sling or polyglactin mesh (15/15)	No sling (15/15)
Zanoni, 2020	Italy	Prospective cohort study	RARP	Bladder neck	retrotrigonal muscular layer	6.05 (3.52)	60.58 (7.71)	24.02 (4.15)	TZ sling (157/157)	No sling (250/250)

Note

BMI, body mass index; NR, not reported; PSA, prostate specific antigen; RARP, robotic assisted radical prostatectomy; RCT, randomized controlled trial; RRP, retropubic radical prostatectomy; SD, standard deviation.

The mean age reported in the studies was 62.06. All studies found were in English. The characteristics of included studies including type of surgical approach are presented in Table 1.

### 3.3 Risk of bias assessment

The risk of bias assessments of the included studies are presented in Tables 2 and 3.

**TABLE 2:** Risk of bias assessment of randomized trials via Cochrane Collaboration's tool for Randomized Controlled Trials

Author, year	Random sequence generation	Allocation concealment	Blinding of participants and personnel	Blinding of outcome assessment	Incomplete outcome data	Selective reporting	Other source of bias
Altinova, 2009	Unclear	Unclear	Unclear	Unclear	Low risk	Low risk	Low risk
Bahler, 2016	Low risk	Low risk	High risk	Unclear	High risk	Low risk	High risk
Nguyen 2017	Unclear	Unclear	High risk	Low risk	Low risk	Low risk	High risk
Cestari 2015	Low risk	Low risk	Unclear	Unclear	Low risk	Low risk	Low risk
Kojima 2014	Unclear	Unclear	High risk	Low risk	Low risk	Low risk	High risk

Notes

Low risk of bias, Low risk of bias present in the study; High risk of bias, High risk of bias present in the study; Unclear, Insufficient information to permit judgment of “Low risk” or “High risk”.

**TABLE 3:** Risk of bias assessment of comparative study via Newcastle‐Ottawa Scale

Author, year	Selection				Comparability		Outcome			Total
	Representativeness of the exposed cohort	Selection of the nonexposed cohort	Ascertainment of exposure	Demonstration that outcome of interest was not present at start of study	Study controls for age	Study controls for any additional factor	Assessment of outcome	Was follow‐up long enough for outcomes to occur	Adequacy of follow up of cohorts	
Jorion, 1997	1	0	1	0	0	0	0	1	1	4
Jones, 2005	1	1	1	0	1	0	1	1	1	7
Westney, 2006	1	1	1	1	0	0	0	1	0	5
Punnen, 2014	1	0	1	1	0	0	0	0	0	3
Zanoni, 2020	1	0	1	1	1	1	0	0	1	6

Note

The score ranges from 0 to 9 points. Study with a score of ≥ 7 was considered as having high quality and low risk of bias.

### 3.4 Cochrane collaboration's tool for randomized controlled trials

Three studies had at least one domain with high risk of bias (Table 2). The most common methodological flaw was found in the “blinding of participants and personnel” and “other source of bias,” which included selection bias and recall bias. Least methodological flaws were found in the domain of “selective reporting.”

### 3.5 Newcastle‐Ottawa scale for comparative studies

One study was assessed to have low risk of bias and four studies had high risk of bias (Table 3). Low score was observed in the domain of comparability in all studies. Risk of bias was lower in the domain of selection and outcome.

### 3.6 Continence outcomes

#### 3.6.1 Continence defined by number of pads^10^, ^18^, ^19^, ^20^, ^22^, ^23^, ^24^, ^26^

**FIGURE 2:**
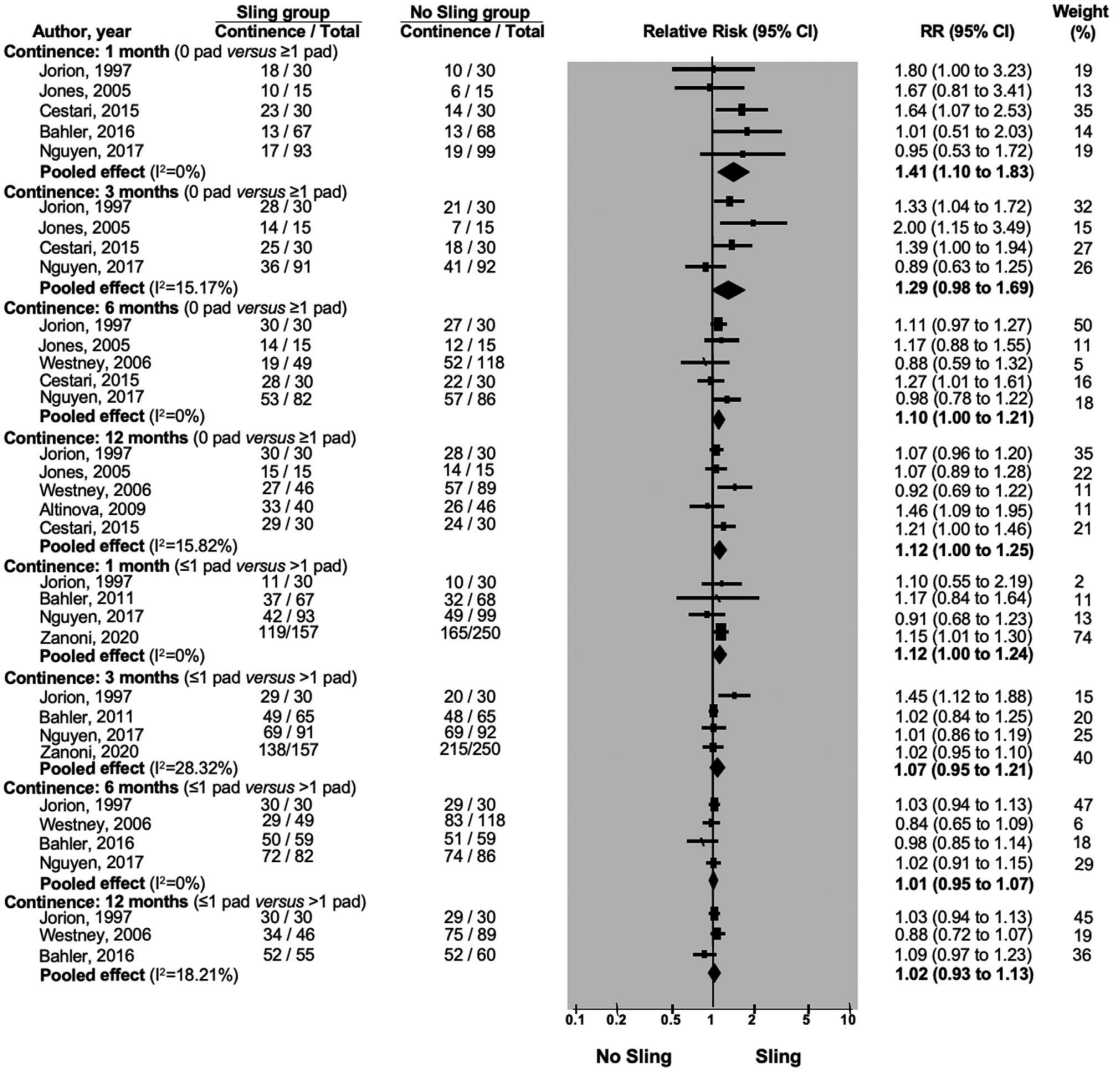
Forrest plot of continence outcomes when continence is defined as using 0 pad versus ≥ 1 pad or as using ≤ 1 pad versus > 1 pad

Our meta‐analysis demonstrated a very‐low to moderate‐quality evidence that with continence defined as using zero pad/day, number of continent patients is significantly increased in the sling group at 1 month (RR:1.41; 95%CI: 1.10 to 1.83), but not at 3 months (RR:1.29; 95%CI: 0.98 to 1.69), 6 months (RR:1.10; 95%CI: 1.00 to 1.21), or 12 months (RR:1.12; 95%CI: 1.00 to 1.25) postoperatively (n = 746) (Figure 2).^10^, ^18^, ^19^, ^20^, ^22^, ^23^, ^24^ However, with continence definition of ≤ 1pad/day, there was a low to moderate‐quality evidence that sling procedures did not reduce the risk of incontinence at 1 month (RR:1.12; 95%CI: 1.00 to 1.24), 3 months (RR:1.07; 95%CI: 0.95 to 1.21), 6 months (RR:1.01; 95%CI: 0.95 to 1.07), and 12 months (RR: 1.02; 95%CI: 0.93 to 1.13) postoperatively (n = 794) (Figure 2).^10^, ^18^, ^22^, ^24^, ^26^

**FIGURE 3:**
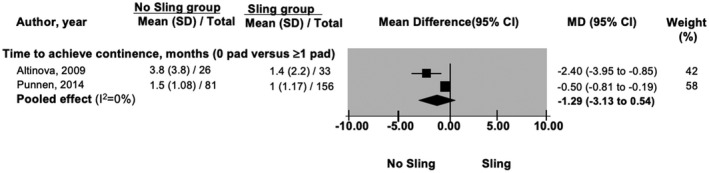
Forrest plot of time taken to achieve continence (months)

Sling procedure did not reduce the time taken to achieve continence with zero pad/day definition^20^, ^25^ (n = 296) (MD: 1.29; 95%CI: −0.54 to 3.13) (Figure 3). Study by Bahler et al. was not included in this meta‐analysis as the study did not report confidence interval for the data, however, the study coincides with our finding^18^ (n = 104) (MD: −0.4; p = 0.61; 95% CI: not reported). However, with ≤ 1pad/day definition, sling group achieved continence 0.5 week earlier than the no‐sling group (MD: 0.5; 95%CI: 0.1 to 0.9).^25^

Furthermore, sling procedure did not reduce the number of pads required per day (n = 60) at 5 days (MD: −0.2; 95%CI: −0.9 to 0.5) and 10 days postoperatively (MD: −0.5; 95% CI: −1.2 to 0.2). Yet, there was a low‐quality evidence demonstrating reduced pad use in the sling group at 1 month (MD: −0.7; 95%CI: −1.2 to −0.2).^19^ There was a low‐quality evidence that sling procedure does not result in more patients achieving immediate continence after catheter removal (n = 59) (RR: 1.31; 95%CI: 0.69 to 2.51).^20^

#### 3.6.2 *Continence defined by pad weight via 1‐hour pad test* ^21^

There is a very‐low‐quality evidence that sling procedure reduces the mean pad weight gain at 1 month (MD: 21.55; 95%CI: 12.58 to 30.52) but not at 3 months (MD: 0.00; 95%CI: −4.12 to 4.12) and 6 months (MD: 0.41; 95%CI: −0.79 to 1.91) postoperatively (n = 57).^21^

#### 3.6.3 Patient reported outcomes measures^21^, ^26^

**FIGURE 4:**
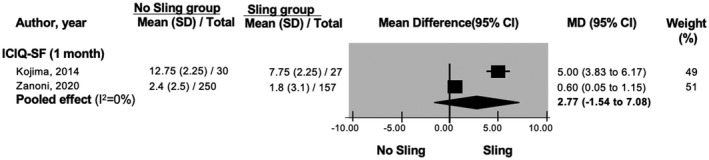
Forrest plot of International Consultation on Incontinence Questionnaire short form score at 1 month

A significant difference in International Consultation on Incontinence Questionnaire short‐form (ICIQ‐SF) favoring the sling group was found at 3 months (MD: 2.25; 95%CI: 1.26 to 3.24) but not at 1 month (MD: 2.77; 95%CI: −1.54 to 7.08) (Figure 4) or 6 months (MD: 0.5; 95%CI: −0.63 to 1.63). For Expanded Prostate Cancer Index Composite (EPIC‐U) results, a significant difference favoring sling procedures is seen at 1 month (MD: 15.35; 95%CI: 11.37 to 19.33), 3 months (MD: 5.30; 95%CI: 1.21 to 9.39) but not at 6 months (MD: 4; 95%CI: −0.04 to 8.04) postoperatively. In addition, a significant difference in International Prostate Symptom Score (IPSS) favoring the sling group was found at 1 month (MD: 4.75; 95%CI: 3.67 to 5.83) and 3 months (MD: 1.44; 95%CI: 0.14 to 2.74) but found to favor the no‐sling group at 6 months (MD: −0.94; 95%CI: −1.77 to −0.11).

#### 3.6.4 Adverse events and complications

**FIGURE 5:**
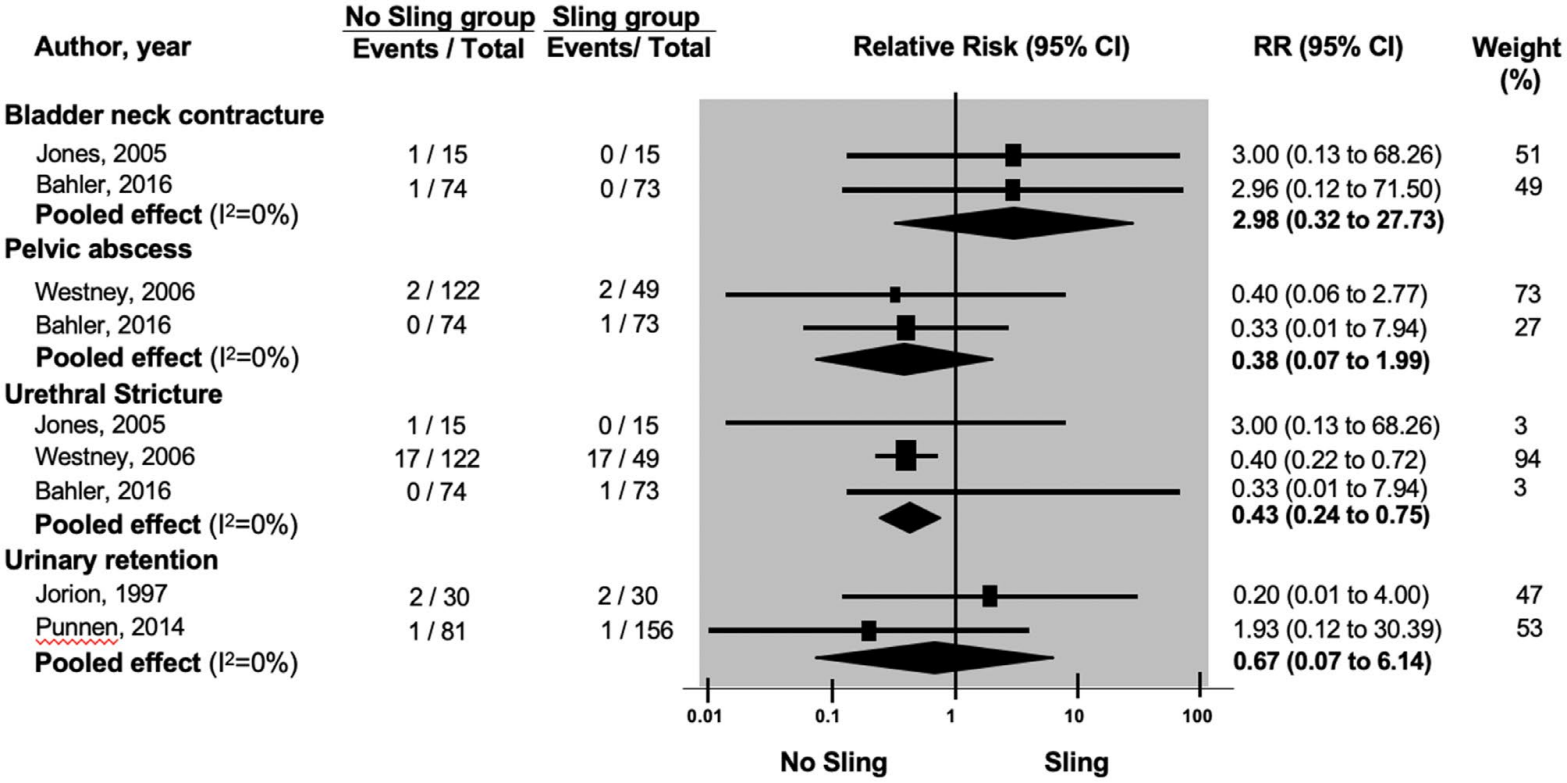
Forrest plot of adverse events

There is a very‐low‐quality evidence that sling does not reduce the incidence of bladder neck contracture (n = 177) (RR: 0.34; 95%CI: 0.04 to 3.12) and moderate‐quality evidence of no effect on pelvic abscess formation (n = 318) (RR; 2.63; 95%CI: 0.50 to 13.70). However, there is a low‐quality evidence of reduced incidence of urethral stricture (n = 348) (RR: 2.35; 95%CI: 1.33 to 4.13) and very‐low‐quality evidence of reduced incidence of urinary retention (n = 297) (RR: 2.09; 95%CI: 1.26 to 3.48)^24^, ^25^ in the sling group (Figure 5).

#### 3.6.5 Length of hospital stay, length of operation, quality of life and cost

**FIGURE 6:**
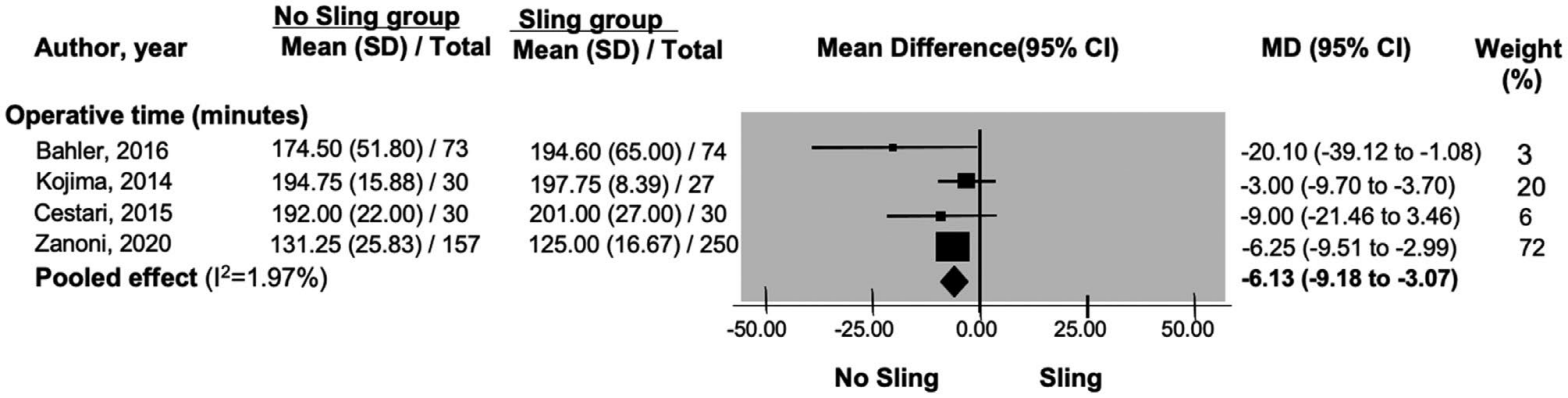
Forrest plot of operative time (minutes)

Cestari et al.^19^ reported that sling procedure does not reduce hospital length of stay (MD: 0.0; *P*‐value not reported; 95% CI not reported) (n = 60). Sling procedure, however, resulted in increase in the length of operation (MD: −6.13, 95%CI: −9.18 to −3.07) (Figure 6). Our search did not find any study investigating differences in quality‐of‐life outcomes and cost.

## 4 DISCUSSION

Our systematic review demonstrates that there is a low‐moderate certainty evidence that intraoperative sling procedures are generally favorable for short‐term improvement but have no difference in long‐term continence outcomes compared to having no sling procedures (Table 4). Early return of continence in the sling group is seen in patient‐reported questionnaires for up to 3 months (IPSS, ICIQ‐SF, and EPIC‐U) postoperatively. Furthermore, significant improvement is seen in the sling group at 1 month for pad weight test, number of pads used per day, and number of continent patients with zero pad/day definition, although no difference is discerned at longer term.

**TABLE 4:** Summary of findings and quality of evidence assessment (GRADE)

Outcome—Time point (*definition*) [references]	Summary of findings		Quality of evidence assessment (GRADE)			
	Number of participants (studies)	Effect size (95%CI)	Study limitation	Inconsistency	Imprecision	Quality
***Continence***						
**Continence**—**1 month** (*0 pad versus ≥ 1 pad*) [Jorion, 1997; Jones, 2005; Cestari, 2015; Bahler, 2016; Nguyen, 2017]	477 (3 RCTs/ 2 comparative studies)	RR: 1.41 (1.10 to 1.83)	−1	−1	None	⨁⨁◯◯
						Low
**Continence**—**3 months** (*0 pad versus ≥ 1 pad*) [Jorion, 1997; Jones, 2005; Cestari, 2015; Nguyen, 2017]	333 (2 RCTs/2 comparative studies)	RR: 1.29 (0.98 to 1.69)	−1	−1	None	⨁⨁◯◯
						Low
**Continence**—**6 months** (*0 pad versus ≥ 1 pad*) [Jorion, 1997; Jones, 2005; Westney, 2006; Cestari, 2015; Nguyen, 2017]	485 (2 RCTs/3 comparative studies)	RR: 1.10 (1.00 to 1.21)	−1	−1	None	⨁⨁◯◯
						Low
**Continence**—**12 months** (*0 pad versus ≥ 1 pad*) [Jorion, 1997; Jones, 2005; Westney, 2006; Altinova, 2009; Cestari, 2015]	371 (2 RCTs/3 comparative studies)	RR: 1.12 (1.00 to 1.25)	−1	−1	None	⨁⨁◯◯
						Low
**Continence**—**1 month** *(≤ 1 pad versus > 1 pad*) [Jorion, 1997; Bahler, 2011; Nguyen, 2017; Zanoni, 2020]	794 (2 RCTs/2 comparative study)	RR: 1.12 (1.00 to 1.24)	−1	−1	None	⨁⨁◯◯
						Low
**Continence**—**3 months** (*≤ 1 pad versus > 1 pad*) [Jorion, 1997; Bahler, 2011; Nguyen, 2017; Zanoni, 2020]	780 (2 RCTs/2 comparative study)	RR: 1.07 (0.95 to 1.21)	−1	None	None	⨁⨁⨁◯
						Moderate
**Continence**—**6 months** *(≤ 1 pad versus > 1 pad*) [Jorion, 1997; Westney, 2006; Bahler, 2011; Nguyen, 2017]	513 (2 RCTs/2 comparative studies)	RR: 1.01 (0.95 to 1.07)	−1	−1	None	⨁⨁◯◯
						Low
**Continence**—**12 months** *(≤ 1 pad versus > 1 pad*) [Jorion, 1997; Westney, 2006; Bahler, 2011]	310 (1 RCT/2 comparative studies)	RR: 1.02 (0.93 to 1.13)	−1	−1	None	⨁⨁◯◯
						Low
**Time to achieve continence,** months (*0 pad versus ≥ 1 pad*) [Altinova, 2009; Punnen, 2014]	296 (1 RCT; 1 comparative study)	MD: −1.29 (−3.13 to 0.54)	−1	−1	−1	⨁◯◯◯
						Very low
**Time to achieve continence**, months (*0 pad versus ≥ 1 pad*) [Bahler, 2011]	104 (1 RCT)	MD: −0.4 (95%CI Not reported), p = 0.61	−1	−1	−1	⨁◯◯◯
						Very low
**Time to achieve continence,** months *(≤ 1 pad versus > 1 pad)* [Punnen, 2014]	237 (1 comparative study)	MD: 0.5 (0.1 to 0.9)	−1	−1	−1	⨁◯◯◯
						Very low
**Number of pads required**—5 days [Cestari, 2015]	60 (1 RCT)	MD: −0.2 (−0.9 to 0.5)	None	−1	−1	⨁⨁◯◯
						Low
**Number of pads required**—10 days [Cestari, 2015]	60 (1 RCT)	MD: −0.5 (−1.2 to 0.2)	None	−1	−1	⨁⨁◯◯
						Low
**Number of pads required**—1 month [Cestari, 2015]	60 (1 RCT)	MD: −0.7 (−1.2 to −0.2)	None	−1	−1	⨁⨁◯◯
						Low
**Achieving immediate continence after catheter removal** (*0 pad versus ≥ 1 pad*) [Altinova, 2009]	59 (1 RCT)	RR: 1.31 (0.69 to 2.51)	None	−1	−1	⨁⨁◯◯
						Low
**1‐hour pad test**—1 month [Kojima, 2014]	57 (1 RCT)	MD: 21.55 (12.58 to 30.52)	−1	−1	−1	⨁◯◯◯
						Very low
**1‐hour pad test**—3 months [Kojima, 2014]	57 (1 RCT)	MD: 0.00 (−4.12 to 4.12)	−1	−1	−1	⨁◯◯◯
						Very low
**1‐hour pad test**—6 months [Kojima, 2014]	57 (1 RCT)	MD: 0.41 (−0.79 to 1.91)	−1	−1	−1	⨁◯◯◯
						Very low
**IPSS**—1 month [Kojima, 2014]	57 (1 RCT)	MD: 4.75 (3.67 to 5.83)	−1	−1	−1	⨁◯◯◯
						Very low
**IPSS**—3 months [Kojima, 2014]	57 (1 RCT)	MD: 1.44 (0.14 to 2.74)	−1	−1	−1	⨁◯◯◯
						Very low
**IPSS**—6 months [Kojima, 2014]	57 (1 RCT)	MD: −0.94 (−1.90 to 0.02)	−1	−1	−1	⨁◯◯◯
						Very low
**ICIQ‐ SF**—1 month [Kojima, 2014; Zanoni, 2020]	464 (1 RCT/ 1 comparative study)	MD: 2.77 (−1.54 to 7.08)	−1	None	None	⨁⨁⨁◯
						Moderate
**ICIQ‐ SF**—3 months [Kojima, 2014]	57 (1 RCT)	MD: 2.25 (1.26 to 3.24)	−1	−1	−1	⨁◯◯◯
						Very low
**ICIQ‐ SF**—6 months [Kojima, 2014]	57 (1 RCT)	MD: 0.5 (−0.63 to 1.63)	−1	−1	−1	⨁◯◯◯
						Very low
**EPIC urinary score**—1 month [Kojima, 2014]	57 (1 RCT)	MD: 15.35 (11.37 to 19.33)	−1	−1	−1	⨁◯◯◯
						Very low
**EPIC urinary score**—3 months [Kojima, 2014]	57 (1 RCT)	MD: 5.30 (1.21 to 9.39)	−1	−1	−1	⨁◯◯◯
						Very low
**EPIC urinary score**—6 months [Kojima, 2014]	57 (1 RCT)	MD: 4 (−0.04 to 8.04)	−1	−1	−1	⨁◯◯◯
						Very low
***Postoperative complications***						
**Bladder neck contracture** [Jones, 2005; Bahler, 2016]	177 (1 RCT/1 comparative study)	RR: 2.98 (0.32 to 27.73)	−1	−1	−1	⨁◯◯◯
						Very low
**Pelvic abscess** [Westney 2006; Bahler, 2016]	318 (1 RCT/1 comparative study)	RR: 0.38 (0.07 to 1.99)	−1	None	None	⨁⨁⨁◯
						Moderate
**Urethral Stricture** (Jones, 2005; Westney 2006; Bahler, 2016)	348 (1 RCT/ 2 comparative studies)	RR: 0.43 (0.24 to 0.75)	−1	−1	None	⨁⨁◯◯
						Low
**Urinary retention** [Jorion, 1997; Punnen, 2014]	297 (2 comparative studies)	RR: 0.67 (0.07 to 6.14)	−1	−1	−1	⨁◯◯◯
						Very low
***Length of hospital stay***						
**Hospital stay length** [Cestari, 2015]	60 (1 RCT)	MD: 0.0; (p = NS)*	None	−1	−1	⨁⨁◯◯
						Low
***Length of operation***						
**Length of operation** [Bahler, 2016; Kojima, 2014; Cestari, 2015; Zanoni, 2020]	671 (2 RCTs/2 comparative studies)	MD: −6.13 (−9.18 to −3.07)	−1	None	None	⨁⨁⨁◯
						Moderate

Note

CI, Confidence interval; RCT, Randomized controlled trials; RR, Relative Risk (value > 1 favors sling); MD, Mean difference (negative values favors sling); IPSS, International Prostate Symptom Score; ICIQ‐SF, International Consultation on Incontinence score short form; EPIC, Expanded Prostate Cancer Index Composite.

* Both groups presented the same mean and SD and therefore MD and 95%CI were zero.

However, sling procedure does not increase the number of continent patients at any time point with ≤ 1 pad/day definition. As such, it is evident that variability in results is introduced by the difference in continence definition and methods. Similarly, sling procedure reduces the time taken to achieve continence with ≤ 1 pad/day definition but not with zero pad/day definition.

Our meta‐analysis also demonstrated that intraoperative sling may reduce the incidence of postoperative complications such as urethral stricture and urinary retention, however, no explanation as to why this is the case was provided in the included studies. Sling procedure increased the operative time but did not affect the length of hospital stay.

### 4.1 Strengths and limitations of the study

The strengths of this systematic review include strict adherence to the Cochrane Collaboration guideline and PRISMA guidelines, registration of the protocol on PROSPERO, utilization of a highly sensitive search strategy with no language and date restriction, inclusion of RCTs, strict assessment of quality with the Cochrane risk of bias tool and Newcastle‐Ottawa Scale and use of the GRADE approach for evidence appraisal.

This review has some limitations. First, intraoperative sling procedure is a relatively new technique in the literature, and thus, only five RCTs were identified and included in our analysis.^10^ Moreover, the inclusion of nonrandomized comparative studies and difficulty in standardizing the tension of the sling, surgical technique, and sling material may have influenced the results. This systematic review also does not include data from ongoing studies, studies published as abstract only and unpublished RCTs. Furthermore, variability in continence measurements resulted in very‐low‐quality evidence of data due to inability to perform meta‐analyses for many continence outcomes.

### 4.2 Comparison with other studies and future directions

To our knowledge, this is the first systematic review and meta‐analysis that evaluates the effectiveness of intraoperative sling procedures compared to having no sling procedures on postprostatectomy continence outcomes. Our review demonstrates the importance of standardizing the definition of continence for utilization in future studies. Although defining continence via pad number is a common clinical practice, it is not a reliable measure of urine leakage as it is largely affected by pad size and type as well as the variability of individual patient's perception of when to change the pads.^27^ Thus, pad weight measurement via 24‐hour pad test is a more objective assessment of urinary incontinence for use in future studies.^27^, ^28^ Additionally, questionnaires are objective assessment tools that also allow the evaluation of patient's postoperative quality of life, and thus, provide a better assessment of sling effectiveness.^27^

Furthermore, our review demonstrates that current studies in the field of intraoperative sling technique have very‐low‐quality to moderate‐quality, identifying the need for future studies with high‐quality evidence.

Our review also suggests that future studies assessing the effectiveness of intraoperative sling must consider the type of sling material used. The sling materials used in all studies included in our review are biological absorbable graft materials, and as they are known to degrade very quickly, this may be one reason for poor outcome. Our review demonstrates that there are no significant difference in continent patients between the sling and no‐sling group beyond 6 months postoperatively. This may be influenced by the fact that biological sling materials are absorbed after 6 months.

## 5 CONCLUSION

Overall, our study demonstrates that intraoperative sling procedures do not decrease long‐term urinary incontinence rate, however, may have potential to promote early return of continence. Currently, our evidence is limited by the lack of high‐quality studies and variability in definitions, and as thus our study details how future clinical research in this field can be improved in order to verify the effect of intraoperative slings more effectively.

## CONFLICTS OF INTEREST

None.

## Supporting information

Table S1

Table S2
