# Supplementary material for: To sling or not to sling? Impact of intraoperative sling procedures during radical prostatectomy on postoperative continence outcomes: A systematic review and meta‐analysis
Source: BJUI Compass. 2021 Jan 17;2(4):226–37. doi: 10.1002/bco2.67 (PMC8988831; doi:10.1002/bco2.67)
Supplement: Supplementary file 1 — Table S1 [file BCO2-2-226-s002.docx]

Supplementary Table 1. Search strategy

| Search Strategy |  |
| --- | --- |
| #1 | Prostate cancer OR Prosta* carcinoma OR Prostat* tumour |
| #2 | Prostatectomy OR radical prostatectomy OR RARP OR MIRP OR RRP OR LRP |
| #3 | Suburethral Sling OR Sling OR suspension OR intraoperative sling |
| #4 | Urinary incontinence OR Incontinen* OR Continen* OR Leak* OR Urin* |
| #1 AND #2 AND #3 AND #4 | |
